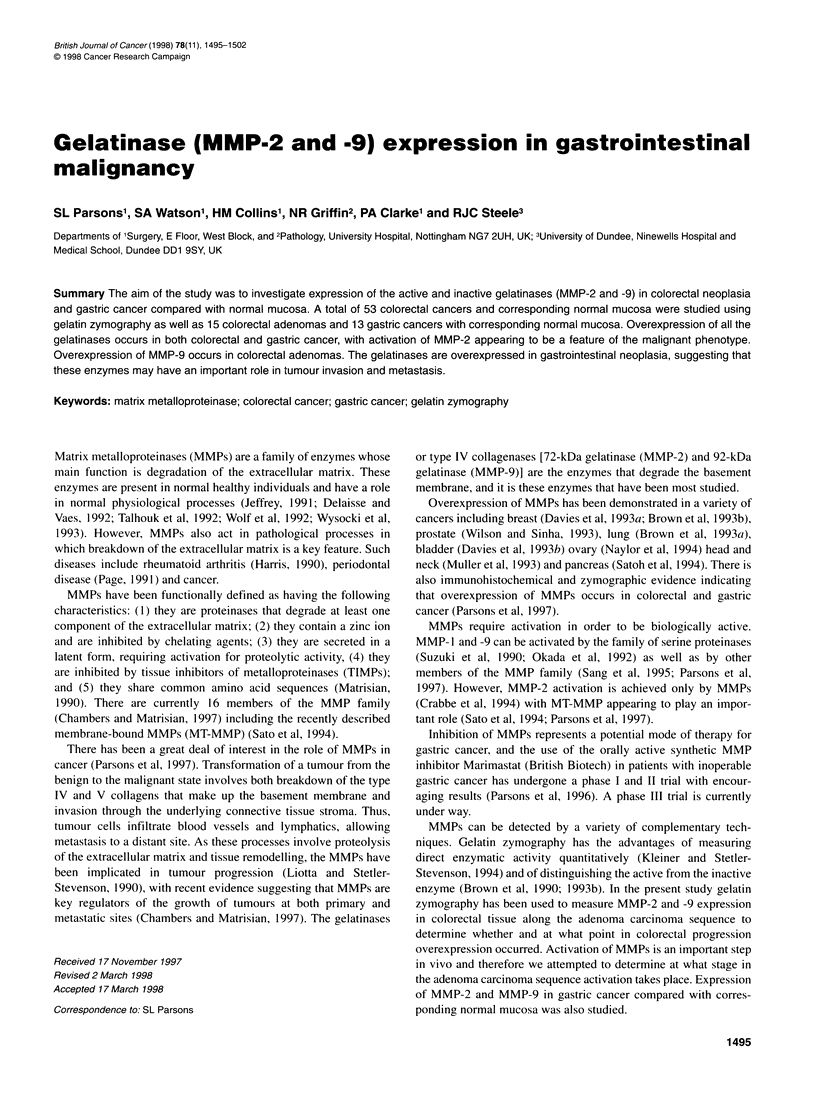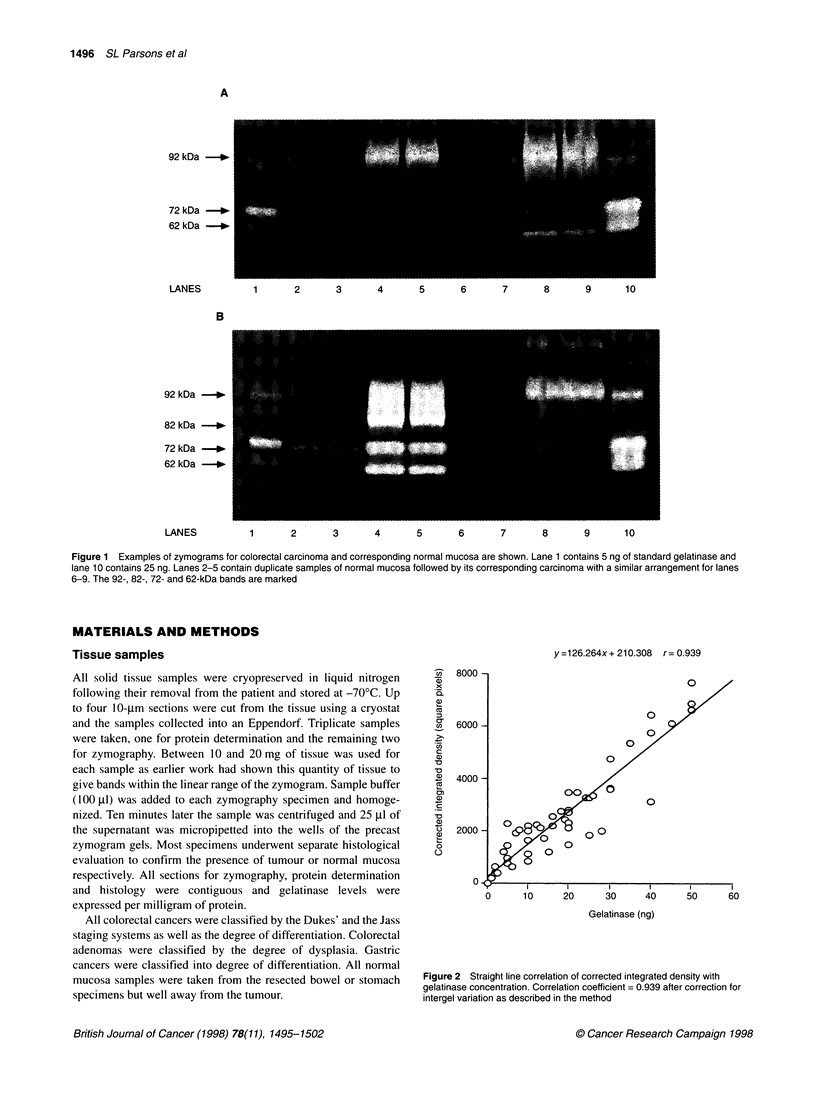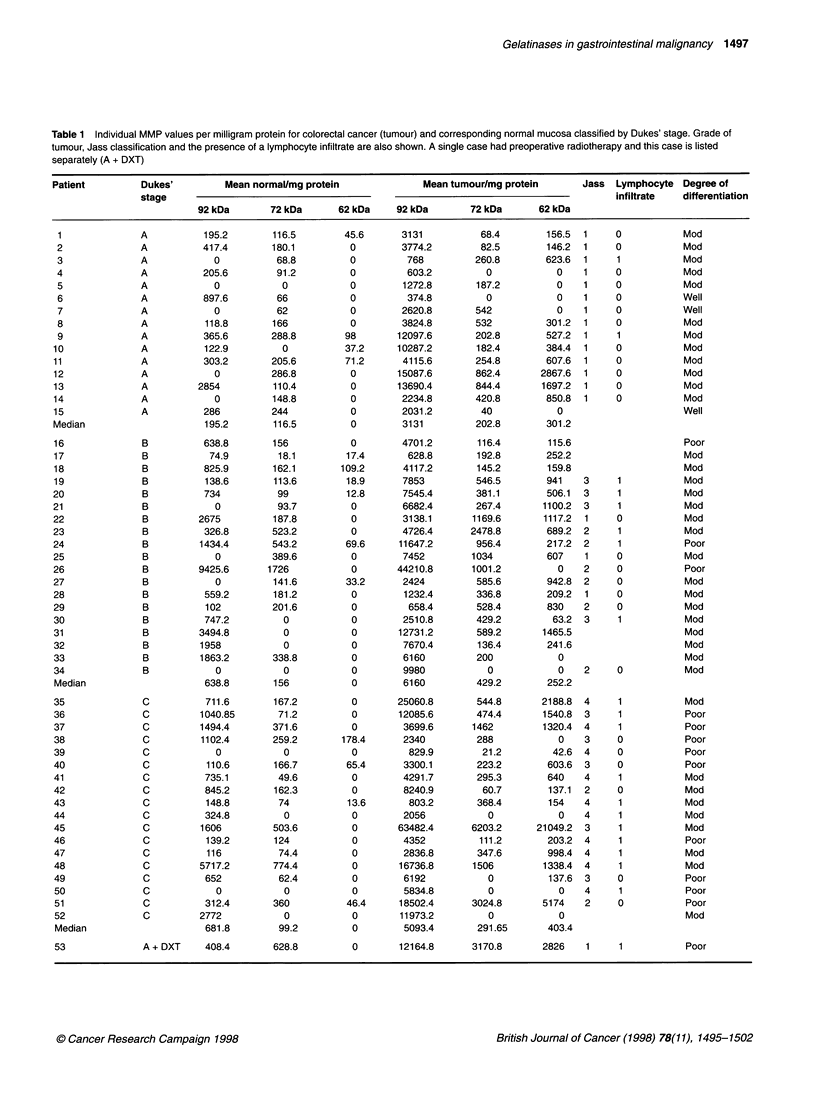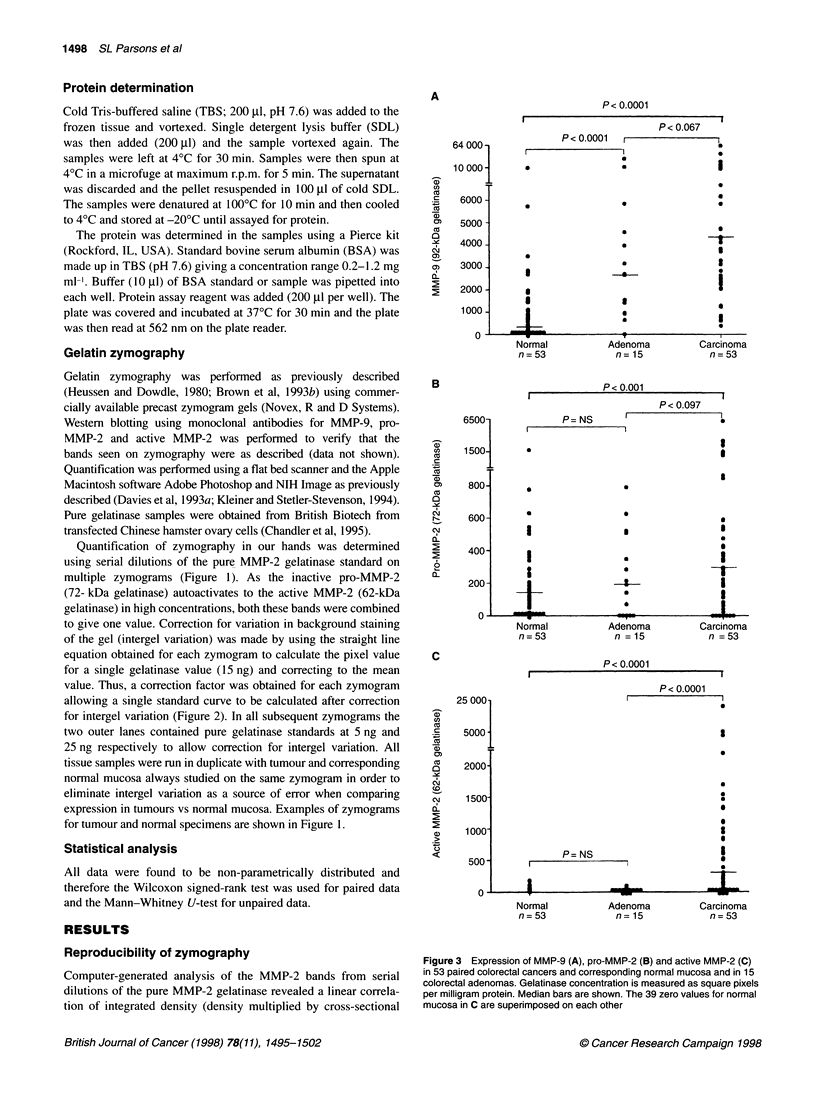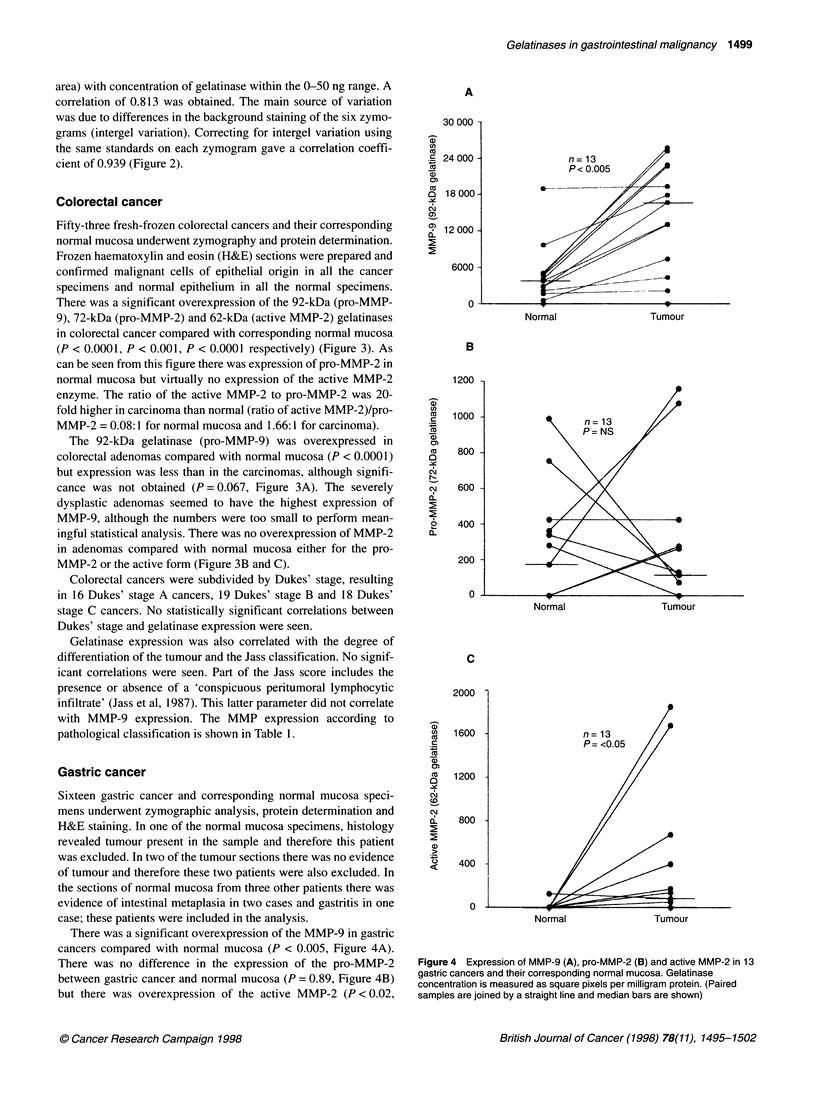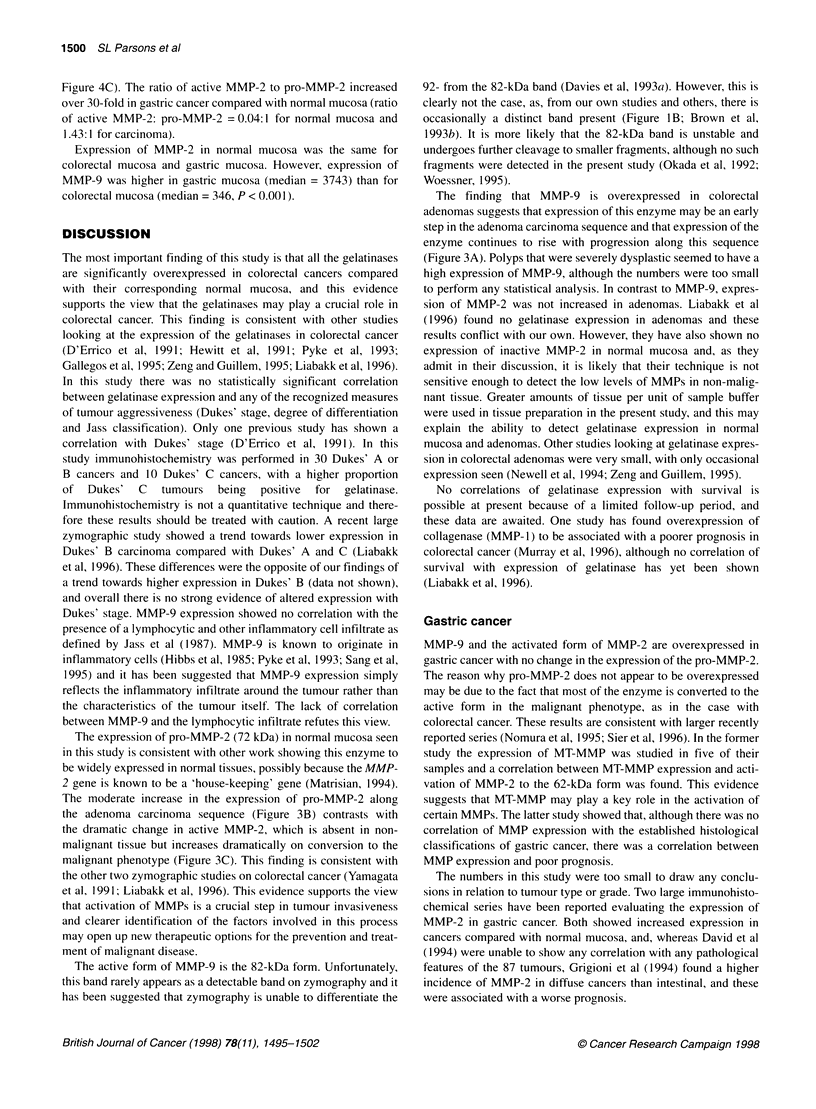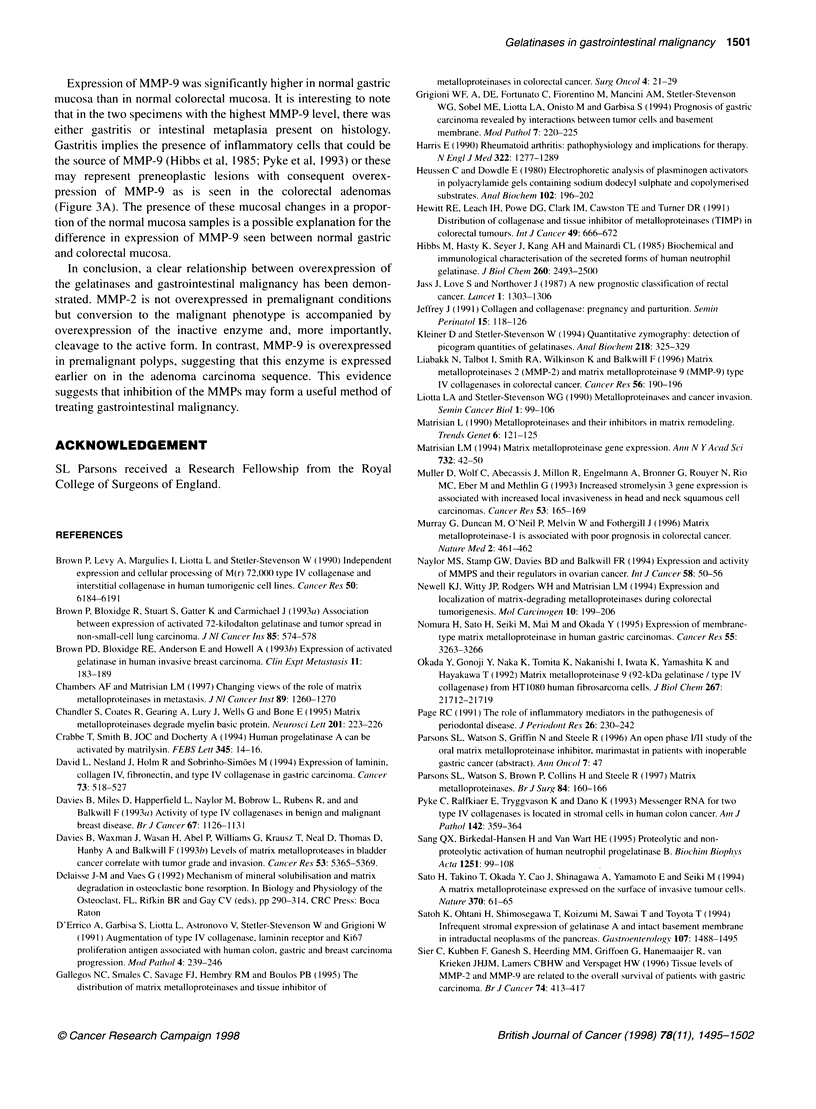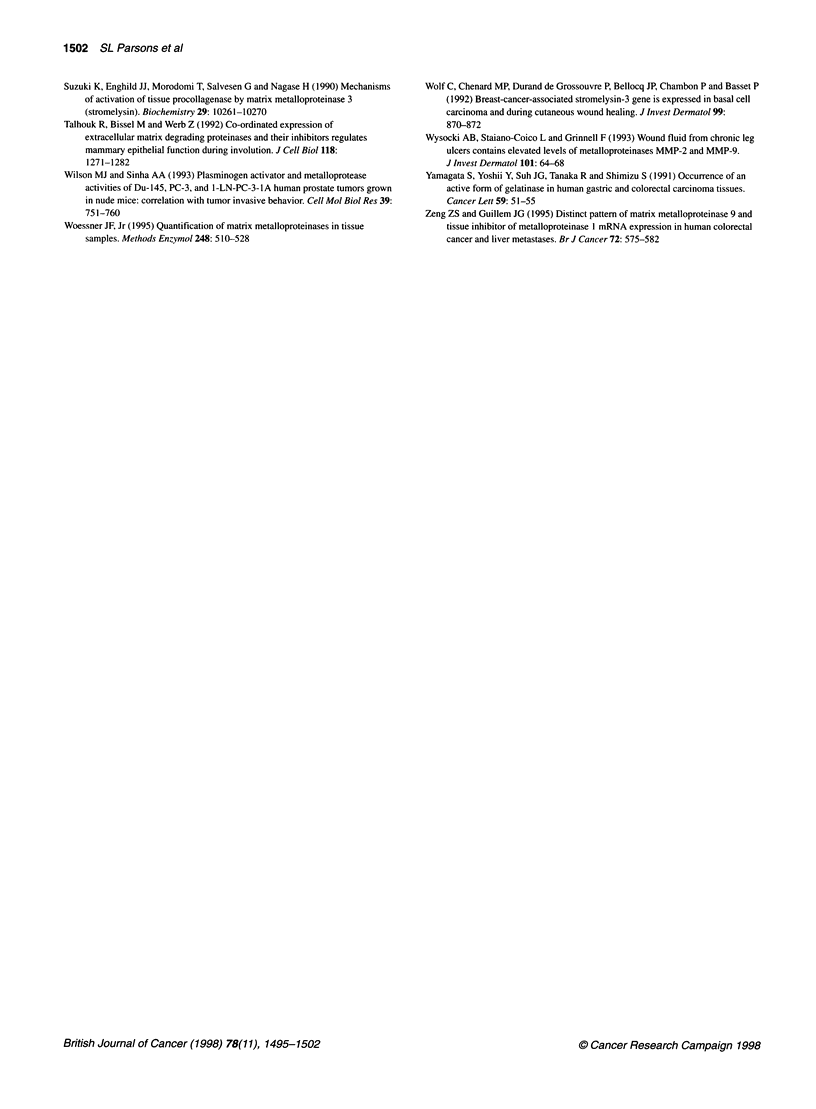# Gelatinase (MMP-2 and -9) expression in gastrointestinal malignancy.

**DOI:** 10.1038/bjc.1998.712

**Published:** 1998-12

**Authors:** S. L. Parsons, S. A. Watson, H. M. Collins, N. R. Griffin, P. A. Clarke, R. J. Steele

**Affiliations:** Department of Surgery, University Hospital, Nottingham, UK.

## Abstract

**Images:**


*British Journal of Cancer* (1998) **78**(11), 1495–1502
© 1998 Cancer Research Campaign

### Gelatinase (MMP-2 and -9) expression in gastrointestinal malignancy

**SL Parsons[1], SA Watson[1], HM Collins[1], NR Griffin[2], PA Clarke[1] and RJC Steele[3]**

Departments of [1]Surgery, E Floor, West Block, and [2]Pathology, University Hospital, Nottingham NG7 2UH, UK; [3]University of Dundee, Ninewells Hospital and Medical School, Dundee DD1 9SY, UK

**Summary** The aim of the study was to investigate expression of the active and inactive gelatinases (MMP-2 and -9) in colorectal neoplasia and gastric cancer compared with normal mucosa. A total of 53 colorectal cancers and corresponding normal mucosa were studied using gelatin zymography as well as 15 colorectal adenomas and 13 gastric cancers with corresponding normal mucosa. Overexpression of all the gelatinases occurs in both colorectal and gastric cancer, with activation of MMP-2 appearing to be a feature of the malignant phenotype. Overexpression of MMP-9 occurs in colorectal adenomas. The gelatinases are overexpressed in gastrointestinal neoplasia, suggesting that these enzymes may have an important role in tumour invasion and metastasis.

**Keywords:** matrix metalloproteinase; colorectal cancer; gastric cancer; gelatin zymography

Matrix metalloproteinases (MMPs) are a family of enzymes whose main function is degradation of the extracellular matrix. These enzymes are present in normal healthy individuals and have a role in normal physiological processes (Jeffrey, 1991; Delaisse and Vaes, 1992; Talhouk et al, 1992; Wolf et al, 1992; Wysocki et al, 1993). However, MMPs also act in pathological processes in which breakdown of the extracellular matrix is a key feature. Such diseases include rheumatoid arthritis (Harris, 1990), periodontal disease (Page, 1991) and cancer.

MMPs have been functionally defined as having the following characteristics: (1) they are proteinases that degrade at least one component of the extracellular matrix; (2) they contain a zinc ion and are inhibited by chelating agents; (3) they are secreted in a latent form, requiring activation for proteolytic activity, (4) they are inhibited by tissue inhibitors of metalloproteinases (TIMPs); and (5) they share common amino acid sequences (Matrisian, 1990). There are currently 16 members of the MMP family (Chambers and Matrisian, 1997) including the recently described membrane-bound MMPs (MT-MMP) (Sato et al, 1994).

There has been a great deal of interest in the role of MMPs in cancer (Parsons et al, 1997). Transformation of a tumour from the benign to the malignant state involves both breakdown of the type IV and V collagens that make up the basement membrane and invasion through the underlying connective tissue stroma. Thus, tumour cells infiltrate blood vessels and lymphatics, allowing metastasis to a distant site. As these processes involve proteolysis of the extracellular matrix and tissue remodelling, the MMPs have been implicated in tumour progression (Liotta and Stetler-Stevenson, 1990), with recent evidence suggesting that MMPs are key regulators of the growth of tumours at both primary and metastatic sites (Chambers and Matrisian, 1997). The gelatinases or type IV collagenases [72-kDa gelatinase (MMP-2) and 92-kDa gelatinase (MMP-9)] are the enzymes that degrade the basement membrane, and it is these enzymes that have been most studied.

Overexpression of MMPs has been demonstrated in a variety of cancers including breast (Davies et al, 1993*a*; Brown et al, 1993b), prostate (Wilson and Sinha, 1993), lung (Brown et al, 1993*a*), bladder (Davies et al, 1993*b*) ovary (Naylor et al, 1994) head and neck (Muller et al, 1993) and pancreas (Satoh et al, 1994). There is also immunohistochemical and zymographic evidence indicating that overexpression of MMPs occurs in colorectal and gastric cancer (Parsons et al, 1997).

MMPs require activation in order to be biologically active. MMP-1 and -9 can be activated by the family of serine proteinases (Suzuki et al, 1990; Okada et al, 1992) as well as by other members of the MMP family (Sang et al, 1995; Parsons et al, 1997). However, MMP-2 activation is achieved only by MMPs (Crabbe et al, 1994) with MT-MMP appearing to play an important role (Sato et al, 1994; Parsons et al, 1997).

Inhibition of MMPs represents a potential mode of therapy for gastric cancer, and the use of the orally active synthetic MMP inhibitor Marimastat (British Biotech) in patients with inoperable gastric cancer has undergone a phase I and II trial with encouraging results (Parsons et al, 1996). A phase III trial is currently under way.

MMPs can be detected by a variety of complementary techniques. Gelatin zymography has the advantages of measuring direct enzymatic activity quantitatively (Kleiner and Stetler-Stevenson, 1994) and of distinguishing the active from the inactive enzyme (Brown et al, 1990; 1993b). In the present study gelatin zymography has been used to measure MMP-2 and -9 expression in colorectal tissue along the adenoma carcinoma sequence to determine whether and at what point in colorectal progression overexpression occurred. Activation of MMPs is an important step in vivo and therefore we attempted to determine at what stage in the adenoma carcinoma sequence activation takes place. Expression of MMP-2 and MMP-9 in gastric cancer compared with corresponding normal mucosa was also studied.

*Received 17 November 1997*
*Revised 2 March 1998*
*Accepted 17 March 1998*

*Correspondence to:* SL Parsons

**Figure 1** Examples of zymograms for colorectal carcinoma and corresponding normal mucosa are shown. Lane 1 contains 5 ng of standard gelatinase and lane 10 contains 25 ng. Lanes 2–5 contain duplicate samples of normal mucosa followed by its corresponding carcinoma with a similar arrangement for lanes 6–9. The 92-, 82-, 72- and 62-kDa bands are marked

### MATERIALS AND METHODS

### Tissue samples

All solid tissue samples were cryopreserved in liquid nitrogen following their removal from the patient and stored at –70°C. Up to four 10-μm sections were cut from the tissue using a cryostat and the samples collected into an Eppendorf. Triplicate samples were taken, one for protein determination and the remaining two for zymography. Between 10 and 20 mg of tissue was used for each sample as earlier work had shown this quantity of tissue to give bands within the linear range of the zymogram. Sample buffer (100 μl) was added to each zymography specimen and homogenized. Ten minutes later the sample was centrifuged and 25 μl of the supernatant was micropipetted into the wells of the precast zymogram gels. Most specimens underwent separate histological evaluation to confirm the presence of tumour or normal mucosa respectively. All sections for zymography, protein determination and histology were contiguous and gelatinase levels were expressed per milligram of protein.

All colorectal cancers were classified by the Dukes' and the Jass staging systems as well as the degree of differentiation. Colorectal adenomas were classified by the degree of dysplasia. Gastric cancers were classified into degree of differentiation. All normal mucosa samples were taken from the resected bowel or stomach specimens but well away from the tumour.

**Figure 2** Straight line correlation of corrected integrated density with gelatinase concentration. Correlation coefficient = 0.939 after correction for intergel variation as described in the method

© Cancer Research Campaign 1998

**Table 1** Individual MMP values per milligram protein for colorectal cancer (tumour) and corresponding normal mucosa classified by Dukes' stage. Grade of tumour, Jass classification and the presence of a lymphocyte infiltrate are also shown. A single case had preoperative radiotherapy and this case is listed separately (A + DXT)

| Patient | Dukes' stage | Mean normal/mg protein | | | Mean tumour/mg protein | | | Jass | Lymphocyte infiltrate | Degree of differentiation |
|---|---|---|---|---|---|---|---|---|---|---|
| | | 92 kDa | 72 kDa | 62 kDa | 92 kDa | 72 kDa | 62 kDa | | | |
| 1 | A | 195.2 | 116.5 | 45.6 | 3131 | 68.4 | 156.5 | 1 | 0 | Mod |
| 2 | A | 417.4 | 180.1 | 0 | 3774.2 | 82.5 | 146.2 | 1 | 0 | Mod |
| 3 | A | 0 | 68.8 | 0 | 768 | 260.8 | 623.6 | 1 | 1 | Mod |
| 4 | A | 205.6 | 91.2 | 0 | 603.2 | 0 | 0 | 1 | 0 | Mod |
| 5 | A | 0 | 0 | 0 | 1272.8 | 187.2 | 0 | 1 | 0 | Mod |
| 6 | A | 897.6 | 66 | 0 | 374.8 | 0 | 0 | 1 | 0 | Well |
| 7 | A | 0 | 62 | 0 | 2620.8 | 542 | 0 | 1 | 0 | Well |
| 8 | A | 118.8 | 166 | 0 | 3824.8 | 532 | 301.2 | 1 | 0 | Mod |
| 9 | A | 365.6 | 288.8 | 98 | 12097.6 | 202.8 | 527.2 | 1 | 1 | Mod |
| 10 | A | 122.9 | 0 | 37.2 | 10287.2 | 182.4 | 384.4 | 1 | 0 | Mod |
| 11 | A | 303.2 | 205.6 | 71.2 | 4115.6 | 254.8 | 607.6 | 1 | 0 | Mod |
| 12 | A | 0 | 286.8 | 0 | 15087.6 | 862.4 | 2867.6 | 1 | 0 | Mod |
| 13 | A | 2854 | 110.4 | 0 | 13690.4 | 844.4 | 1697.2 | 1 | 0 | Mod |
| 14 | A | 0 | 148.8 | 0 | 2234.8 | 420.8 | 850.8 | 1 | 0 | Mod |
| 15 | A | 286 | 244 | 0 | 2031.2 | 40 | 0 | | | Well |
| Median | | 195.2 | 116.5 | 0 | 3131 | 202.8 | 301.2 | | | |
| 16 | B | 638.8 | 156 | 0 | 4701.2 | 116.4 | 115.6 | | | Poor |
| 17 | B | 74.9 | 18.1 | 17.4 | 628.8 | 192.8 | 252.2 | | | Mod |
| 18 | B | 825.9 | 162.1 | 109.2 | 4117.2 | 145.2 | 159.8 | | | Mod |
| 19 | B | 138.6 | 113.6 | 18.9 | 7853 | 546.5 | 941 | 3 | 1 | Mod |
| 20 | B | 734 | 99 | 12.8 | 7545.4 | 381.1 | 506.1 | 3 | 1 | Mod |
| 21 | B | 0 | 93.7 | 0 | 6682.4 | 267.4 | 1100.2 | 3 | 1 | Mod |
| 22 | B | 2675 | 187.8 | 0 | 3138.1 | 1169.6 | 1117.2 | 1 | 0 | Mod |
| 23 | B | 326.8 | 523.2 | 0 | 4726.4 | 2478.8 | 689.2 | 2 | 1 | Mod |
| 24 | B | 1434.4 | 543.2 | 69.6 | 11647.2 | 956.4 | 217.2 | 2 | 1 | Poor |
| 25 | B | 0 | 389.6 | 0 | 7452 | 1034 | 607 | 1 | 0 | Mod |
| 26 | B | 9425.6 | 1726 | 0 | 44210.8 | 1001.2 | 0 | 2 | 0 | Poor |
| 27 | B | 0 | 141.6 | 33.2 | 2424 | 585.6 | 942.8 | 2 | 0 | Mod |
| 28 | B | 559.2 | 181.2 | 0 | 1232.4 | 336.8 | 209.2 | 1 | 0 | Mod |
| 29 | B | 102 | 201.6 | 0 | 658.4 | 528.4 | 830 | 2 | 0 | Mod |
| 30 | B | 747.2 | 0 | 0 | 2510.8 | 429.2 | 63.2 | 3 | 1 | Mod |
| 31 | B | 3494.8 | 0 | 0 | 12731.2 | 589.2 | 1465.5 | | | Mod |
| 32 | B | 1958 | 0 | 0 | 7670.4 | 136.4 | 241.6 | | | Mod |
| 33 | B | 1863.2 | 338.8 | 0 | 6160 | 200 | 0 | | | Mod |
| 34 | B | 0 | 0 | 0 | 9980 | 0 | 0 | 2 | 0 | Mod |
| Median | | 638.8 | 156 | 0 | 6160 | 429.2 | 252.2 | | | |
| 35 | C | 711.6 | 167.2 | 0 | 25060.8 | 544.8 | 2188.8 | 4 | 1 | Mod |
| 36 | C | 1040.85 | 71.2 | 0 | 12085.6 | 474.4 | 1540.8 | 3 | 1 | Poor |
| 37 | C | 1494.4 | 371.6 | 0 | 3699.6 | 1462 | 1320.4 | 4 | 1 | Poor |
| 38 | C | 1102.4 | 259.2 | 178.4 | 2340 | 288 | 0 | 3 | 0 | Poor |
| 39 | C | 0 | 0 | 0 | 829.9 | 21.2 | 42.6 | 4 | 0 | Poor |
| 40 | C | 110.6 | 166.7 | 65.4 | 3300.1 | 223.2 | 603.6 | 3 | 0 | Poor |
| 41 | C | 735.1 | 49.6 | 0 | 4291.7 | 295.3 | 640 | 4 | 1 | Mod |
| 42 | C | 845.2 | 162.3 | 0 | 8240.9 | 60.7 | 137.1 | 2 | 0 | Mod |
| 43 | C | 148.8 | 74 | 13.6 | 803.2 | 368.4 | 154 | 4 | 1 | Mod |
| 44 | C | 324.8 | 0 | 0 | 2056 | 0 | 0 | 4 | 1 | Mod |
| 45 | C | 1606 | 503.6 | 0 | 63482.4 | 6203.2 | 21049.2 | 3 | 1 | Mod |
| 46 | C | 139.2 | 124 | 0 | 4352 | 111.2 | 203.2 | 4 | 1 | Poor |
| 47 | C | 116 | 74.4 | 0 | 2836.8 | 347.6 | 998.4 | 4 | 1 | Mod |
| 48 | C | 5717.2 | 774.4 | 0 | 16736.8 | 1506 | 1338.4 | 4 | 1 | Mod |
| 49 | C | 652 | 62.4 | 0 | 6192 | 0 | 137.6 | 3 | 0 | Poor |
| 50 | C | 0 | 0 | 0 | 5834.8 | 0 | 0 | 4 | 1 | Poor |
| 51 | C | 312.4 | 360 | 46.4 | 18502.4 | 3024.8 | 5174 | 2 | 0 | Poor |
| 52 | C | 2772 | 0 | 0 | 11973.2 | 0 | 0 | | | Mod |
| Median | | 681.8 | 99.2 | 0 | 5093.4 | 291.65 | 403.4 | | | |
| 53 | A + DXT | 408.4 | 628.8 | 0 | 12164.8 | 3170.8 | 2826 | 1 | 1 | Poor |

© Cancer Research Campaign 1998

### Protein determination

Cold Tris-buffered saline (TBS; 200 μl, pH 7.6) was added to the frozen tissue and vortexed. Single detergent lysis buffer (SDL) was then added (200 μl) and the sample vortexed again. The samples were left at 4°C for 30 min. Samples were then spun at 4°C in a microfuge at maximum r.p.m. for 5 min. The supernatant was discarded and the pellet resuspended in 100 μl of cold SDL. The samples were denatured at 100°C for 10 min and then cooled to 4°C and stored at –20°C until assayed for protein.

The protein was determined in the samples using a Pierce kit (Rockford, IL, USA). Standard bovine serum albumin (BSA) was made up in TBS (pH 7.6) giving a concentration range 0.2–1.2 mg $ml^{-1}$. Buffer (10 μl) of BSA standard or sample was pipetted into each well. Protein assay reagent was added (200 μl per well). The plate was covered and incubated at 37°C for 30 min and the plate was then read at 562 nm on the plate reader.

### Gelatin zymography

Gelatin zymography was performed as previously described (Heussen and Dowdle, 1980; Brown et al, 1993*b*) using commercially available precast zymogram gels (Novex, R and D Systems). Western blotting using monoclonal antibodies for MMP-9, pro-MMP-2 and active MMP-2 was performed to verify that the bands seen on zymography were as described (data not shown). Quantification was performed using a flat bed scanner and the Apple Macintosh software Adobe Photoshop and NIH Image as previously described (Davies et al, 1993*a*; Kleiner and Stetler-Stevenson, 1994). Pure gelatinase samples were obtained from British Biotech from transfected Chinese hamster ovary cells (Chandler et al, 1995).

Quantification of zymography in our hands was determined using serial dilutions of the pure MMP-2 gelatinase standard on multiple zymograms (Figure 1). As the inactive pro-MMP-2 (72- kDa gelatinase) autoactivates to the active MMP-2 (62-kDa gelatinase) in high concentrations, both these bands were combined to give one value. Correction for variation in background staining of the gel (intergel variation) was made by using the straight line equation obtained for each zymogram to calculate the pixel value for a single gelatinase value (15 ng) and correcting to the mean value. Thus, a correction factor was obtained for each zymogram allowing a single standard curve to be calculated after correction for intergel variation (Figure 2). In all subsequent zymograms the two outer lanes contained pure gelatinase standards at 5 ng and 25 ng respectively to allow correction for intergel variation. All tissue samples were run in duplicate with tumour and corresponding normal mucosa always studied on the same zymogram in order to eliminate intergel variation as a source of error when comparing expression in tumours vs normal mucosa. Examples of zymograms for tumour and normal specimens are shown in Figure 1.

### Statistical analysis

All data were found to be non-parametrically distributed and therefore the Wilcoxon signed-rank test was used for paired data and the Mann–Whitney *U*-test for unpaired data.

### RESULTS

### Reproducibility of zymography

Computer-generated analysis of the MMP-2 bands from serial dilutions of the pure MMP-2 gelatinase revealed a linear correlation of integrated density (density multiplied by cross-sectional

**Figure 3** Expression of MMP-9 (**A**), pro-MMP-2 (**B**) and active MMP-2 (**C**) in 53 paired colorectal cancers and corresponding normal mucosa and in 15 colorectal adenomas. Gelatinase concentration is measured as square pixels per milligram protein. Median bars are shown. The 39 zero values for normal mucosa in **C** are superimposed on each other

© Cancer Research Campaign 1998

area) with concentration of gelatinase within the 0–50 ng range. A correlation of 0.813 was obtained. The main source of variation was due to differences in the background staining of the six zymograms (intergel variation). Correcting for intergel variation using the same standards on each zymogram gave a correlation coefficient of 0.939 (Figure 2).

### Colorectal cancer

Fifty-three fresh-frozen colorectal cancers and their corresponding normal mucosa underwent zymography and protein determination. Frozen haematoxylin and eosin (H&E) sections were prepared and confirmed malignant cells of epithelial origin in all the cancer specimens and normal epithelium in all the normal specimens. There was a significant overexpression of the 92-kDa (pro-MMP-9), 72-kDa (pro-MMP-2) and 62-kDa (active MMP-2) gelatinases in colorectal cancer compared with corresponding normal mucosa ($P < 0.0001$, $P < 0.001$, $P < 0.0001$ respectively) (Figure 3). As can be seen from this figure there was expression of pro-MMP-2 in normal mucosa but virtually no expression of the active MMP-2 enzyme. The ratio of the active MMP-2 to pro-MMP-2 was 20-fold higher in carcinoma than normal (ratio of active MMP-2)/pro-MMP-2 = 0.08:1 for normal mucosa and 1.66:1 for carcinoma).

The 92-kDa gelatinase (pro-MMP-9) was overexpressed in colorectal adenomas compared with normal mucosa ($P < 0.0001$) but expression was less than in the carcinomas, although significance was not obtained ($P = 0.067$, Figure 3A). The severely dysplastic adenomas seemed to have the highest expression of MMP-9, although the numbers were too small to perform meaningful statistical analysis. There was no overexpression of MMP-2 in adenomas compared with normal mucosa either for the pro-MMP-2 or the active form (Figure 3B and C).

Colorectal cancers were subdivided by Dukes' stage, resulting in 16 Dukes' stage A cancers, 19 Dukes' stage B and 18 Dukes' stage C cancers. No statistically significant correlations between Dukes' stage and gelatinase expression were seen.

Gelatinase expression was also correlated with the degree of differentiation of the tumour and the Jass classification. No significant correlations were seen. Part of the Jass score includes the presence or absence of a 'conspicuous peritumoral lymphocytic infiltrate' (Jass et al, 1987). This latter parameter did not correlate with MMP-9 expression. The MMP expression according to pathological classification is shown in Table 1.

### Gastric cancer

Sixteen gastric cancer and corresponding normal mucosa specimens underwent zymographic analysis, protein determination and H&E staining. In one of the normal mucosa specimens, histology revealed tumour present in the sample and therefore this patient was excluded. In two of the tumour sections there was no evidence of tumour and therefore these two patients were also excluded. In the sections of normal mucosa from three other patients there was evidence of intestinal metaplasia in two cases and gastritis in one case; these patients were included in the analysis.

There was a significant overexpression of the MMP-9 in gastric cancers compared with normal mucosa ($P < 0.005$, Figure 4A). There was no difference in the expression of the pro-MMP-2 between gastric cancer and normal mucosa ($P = 0.89$, Figure 4B) but there was overexpression of the active MMP-2 ($P < 0.02$,

**Figure 4** Expression of MMP-9 (**A**), pro-MMP-2 (**B**) and active MMP-2 in 13 gastric cancers and their corresponding normal mucosa. Gelatinase concentration is measured as square pixels per milligram protein. (Paired samples are joined by a straight line and median bars are shown)

© Cancer Research Campaign 1998

Figure 4C). The ratio of active MMP-2 to pro-MMP-2 increased over 30-fold in gastric cancer compared with normal mucosa (ratio of active MMP-2: pro-MMP-2 = 0.04:1 for normal mucosa and 1.43:1 for carcinoma).

Expression of MMP-2 in normal mucosa was the same for colorectal mucosa and gastric mucosa. However, expression of MMP-9 was higher in gastric mucosa (median = 3743) than for colorectal mucosa (median = 346, $P < 0.001$).

### DISCUSSION

The most important finding of this study is that all the gelatinases are significantly overexpressed in colorectal cancers compared with their corresponding normal mucosa, and this evidence supports the view that the gelatinases may play a crucial role in colorectal cancer. This finding is consistent with other studies looking at the expression of the gelatinases in colorectal cancer (D'Errico et al, 1991; Hewitt et al, 1991; Pyke et al, 1993; Gallegos et al, 1995; Zeng and Guillem, 1995; Liabakk et al, 1996). In this study there was no statistically significant correlation between gelatinase expression and any of the recognized measures of tumour aggressiveness (Dukes' stage, degree of differentiation and Jass classification). Only one previous study has shown a correlation with Dukes' stage (D'Errico et al, 1991). In this study immunohistochemistry was performed in 30 Dukes' A or B cancers and 10 Dukes' C cancers, with a higher proportion of Dukes' C tumours being positive for gelatinase. Immunohistochemistry is not a quantitative technique and therefore these results should be treated with caution. A recent large zymographic study showed a trend towards lower expression in Dukes' B carcinoma compared with Dukes' A and C (Liabakk et al, 1996). These differences were the opposite of our findings of a trend towards higher expression in Dukes' B (data not shown), and overall there is no strong evidence of altered expression with Dukes' stage. MMP-9 expression showed no correlation with the presence of a lymphocytic and other inflammatory cell infiltrate as defined by Jass et al (1987). MMP-9 is known to originate in inflammatory cells (Hibbs et al, 1985; Pyke et al, 1993; Sang et al, 1995) and it has been suggested that MMP-9 expression simply reflects the inflammatory infiltrate around the tumour rather than the characteristics of the tumour itself. The lack of correlation between MMP-9 and the lymphocytic infiltrate refutes this view.

The expression of pro-MMP-2 (72 kDa) in normal mucosa seen in this study is consistent with other work showing this enzyme to be widely expressed in normal tissues, possibly because the *MMP-2* gene is known to be a 'house-keeping' gene (Matrisian, 1994). The moderate increase in the expression of pro-MMP-2 along the adenoma carcinoma sequence (Figure 3B) contrasts with the dramatic change in active MMP-2, which is absent in non-malignant tissue but increases dramatically on conversion to the malignant phenotype (Figure 3C). This finding is consistent with the other two zymographic studies on colorectal cancer (Yamagata et al, 1991; Liabakk et al, 1996). This evidence supports the view that activation of MMPs is a crucial step in tumour invasiveness and clearer identification of the factors involved in this process may open up new therapeutic options for the prevention and treatment of malignant disease.

The active form of MMP-9 is the 82-kDa form. Unfortunately, this band rarely appears as a detectable band on zymography and it has been suggested that zymography is unable to differentiate the 92- from the 82-kDa band (Davies et al, 1993*a*). However, this is clearly not the case, as, from our own studies and others, there is occasionally a distinct band present (Figure 1B; Brown et al, 1993*b*). It is more likely that the 82-kDa band is unstable and undergoes further cleavage to smaller fragments, although no such fragments were detected in the present study (Okada et al, 1992; Woessner, 1995).

The finding that MMP-9 is overexpressed in colorectal adenomas suggests that expression of this enzyme may be an early step in the adenoma carcinoma sequence and that expression of the enzyme continues to rise with progression along this sequence (Figure 3A). Polyps that were severely dysplastic seemed to have a high expression of MMP-9, although the numbers were too small to perform any statistical analysis. In contrast to MMP-9, expression of MMP-2 was not increased in adenomas. Liabakk et al (1996) found no gelatinase expression in adenomas and these results conflict with our own. However, they have also shown no expression of inactive MMP-2 in normal mucosa and, as they admit in their discussion, it is likely that their technique is not sensitive enough to detect the low levels of MMPs in non-malignant tissue. Greater amounts of tissue per unit of sample buffer were used in tissue preparation in the present study, and this may explain the ability to detect gelatinase expression in normal mucosa and adenomas. Other studies looking at gelatinase expression in colorectal adenomas were very small, with only occasional expression seen (Newell et al, 1994; Zeng and Guillem, 1995).

No correlations of gelatinase expression with survival is possible at present because of a limited follow-up period, and these data are awaited. One study has found overexpression of collagenase (MMP-1) to be associated with a poorer prognosis in colorectal cancer (Murray et al, 1996), although no correlation of survival with expression of gelatinase has yet been shown (Liabakk et al, 1996).

### Gastric cancer

MMP-9 and the activated form of MMP-2 are overexpressed in gastric cancer with no change in the expression of the pro-MMP-2. The reason why pro-MMP-2 does not appear to be overexpressed may be due to the fact that most of the enzyme is converted to the active form in the malignant phenotype, as in the case with colorectal cancer. These results are consistent with larger recently reported series (Nomura et al, 1995; Sier et al, 1996). In the former study the expression of MT-MMP was studied in five of their samples and a correlation between MT-MMP expression and activation of MMP-2 to the 62-kDa form was found. This evidence suggests that MT-MMP may play a key role in the activation of certain MMPs. The latter study showed that, although there was no correlation of MMP expression with the established histological classifications of gastric cancer, there was a correlation between MMP expression and poor prognosis.

The numbers in this study were too small to draw any conclusions in relation to tumour type or grade. Two large immunohistochemical series have been reported evaluating the expression of MMP-2 in gastric cancer. Both showed increased expression in cancers compared with normal mucosa, and, whereas David et al (1994) were unable to show any correlation with any pathological features of the 87 tumours, Grigioni et al (1994) found a higher incidence of MMP-2 in diffuse cancers than intestinal, and these were associated with a worse prognosis.

*© Cancer Research Campaign 1998*

Expression of MMP-9 was significantly higher in normal gastric mucosa than in normal colorectal mucosa. It is interesting to note that in the two specimens with the highest MMP-9 level, there was either gastritis or intestinal metaplasia present on histology. Gastritis implies the presence of inflammatory cells that could be the source of MMP-9 (Hibbs et al, 1985; Pyke et al, 1993) or these may represent preneoplastic lesions with consequent overexpression of MMP-9 as is seen in the colorectal adenomas (Figure 3A). The presence of these mucosal changes in a proportion of the normal mucosa samples is a possible explanation for the difference in expression of MMP-9 seen between normal gastric and colorectal mucosa.

In conclusion, a clear relationship between overexpression of the gelatinases and gastrointestinal malignancy has been demonstrated. MMP-2 is not overexpressed in premalignant conditions but conversion to the malignant phenotype is accompanied by overexpression of the inactive enzyme and, more importantly, cleavage to the active form. In contrast, MMP-9 is overexpressed in premalignant polyps, suggesting that this enzyme is expressed earlier on in the adenoma carcinoma sequence. This evidence suggests that inhibition of the MMPs may form a useful method of treating gastrointestinal malignancy.

### ACKNOWLEDGEMENT

SL Parsons received a Research Fellowship from the Royal College of Surgeons of England.

### REFERENCES

Brown P, Levy A, Margulies I, Liotta L and Stetler-Stevenson W (1990) Independent expression and cellular processing of M(r) 72,000 type IV collagenase and interstitial collagenase in human tumorigenic cell lines. *Cancer Res* **50**: 6184–6191

Brown P, Bloxidge R, Stuart S, Gatter K and Carmichael J (1993*a*) Association between expression of activated 72-kilodalton gelatinase and tumor spread in non-small-cell lung carcinoma. *J Nl Cancer Ins* **85**: 574–578

Brown PD, Bloxidge RE, Anderson E and Howell A (1993*b*) Expression of activated gelatinase in human invasive breast carcinoma. *Clin Expt Metastasis* **11**: 183–189

Chambers AF and Matrisian LM (1997) Changing views of the role of matrix metalloproteinases in metastasis. *J Nl Cancer Inst* **89**: 1260–1270

Chandler S, Coates R, Gearing A, Lury J, Wells G and Bone E (1995) Matrix metalloproteinases degrade myelin basic protein. *Neurosci Lett* **201**: 223–226

Crabbe T, Smith B, JOC and Docherty A (1994) Human progelatinase A can be activated by matrilysin. *FEBS Lett* **345**: 14–16.

David L, Nesland J, Holm R and Sobrinho-Simões M (1994) Expression of laminin, collagen IV, fibronectin, and type IV collagenase in gastric carcinoma. *Cancer* **73**: 518–527

Davies B, Miles D, Happerfield L, Naylor M, Bobrow L, Rubens R, and and Balkwill F (1993*a*) Activity of type IV collagenases in benign and malignant breast disease. *Br J Cancer* **67**: 1126–1131

Davies B, Waxman J, Wasan H, Abel P, Williams G, Krausz T, Neal D, Thomas D, Hanby A and Balkwill F (1993*b*) Levels of matrix metalloproteases in bladder cancer correlate with tumor grade and invasion. *Cancer Res* **53**: 5365–5369.

Delaisse J-M and Vaes G (1992) Mechanism of mineral solubilisation and matrix degradation in osteoclastic bone resorption. In Biology and Physiology of the Osteoclast, FL, Rifkin BR and Gay CV (eds), pp 290–314, CRC Press: Boca Raton

D'Errico A, Garbisa S, Liotta L, Astronovo V, Stetler-Stevenson W and Grigioni W (1991) Augmentation of type IV collagenase, laminin receptor and Ki67 proliferation antigen associated with human colon, gastric and breast carcinoma progression. *Mod Pathol* **4**: 239–246

Gallegos NC, Smales C, Savage FJ, Hembry RM and Boulos PB (1995) The distribution of matrix metalloproteinases and tissue inhibitor of metalloproteinases in colorectal cancer. *Surg Oncol* **4**: 21–29

Grigioni WF, A, DE, Fortunato C, Fiorentino M, Mancini AM, Stetler-Stevenson WG, Sobel ME, Liotta LA, Onisto M and Garbisa S (1994) Prognosis of gastric carcinoma revealed by interactions between tumor cells and basement membrane. *Mod Pathol* **7**: 220–225

Harris E (1990) Rheumatoid arthritis: pathophysiology and implications for therapy. *N Engl J Med* **322**: 1277–1289

Heussen C and Dowdle E (1980) Electrophoretic analysis of plasminogen activators in polyacrylamide gels containing sodium dodecyl sulphate and copolymerised substrates. *Anal Biochem* **102**: 196–202

Hewitt RE, Leach IH, Powe DG, Clark IM, Cawston TE and Turner DR (1991) Distribution of collagenase and tissue inhibitor of metalloproteinases (TIMP) in colorectal tumours. *Int J Cancer* **49**: 666–672

Hibbs M, Hasty K, Seyer J, Kang AH and Mainardi CL (1985) Biochemical and immunological characterisation of the secreted forms of human neutrophil gelatinase. *J Biol Chem* **260**: 2493–2500

Jass J, Love S and Northover J (1987) A new prognostic classification of rectal cancer. *Lancet* **1**: 1303–1306

Jeffrey J (1991) Collagen and collagenase: pregnancy and parturition. *Semin Perinatol* **15**: 118–126

Kleiner D and Stetler-Stevenson W (1994) Quantitative zymography: detection of picogram quantities of gelatinases. *Anal Biochem* **218**: 325–329

Liabakk N, Talbot I, Smith RA, Wilkinson K and Balkwill F (1996) Matrix metalloproteinases 2 (MMP-2) and matrix metalloproteinase 9 (MMP-9) type IV collagenases in colorectal cancer. *Cancer Res* **56**: 190–196

Liotta LA and Stetler-Stevenson WG (1990) Metalloproteinases and cancer invasion. *Semin Cancer Biol* **1**: 99–106

Matrisian L (1990) Metalloproteinases and their inhibitors in matrix remodeling. *Trends Genet* **6**: 121–125

Matrisian LM (1994) Matrix metalloproteinase gene expression. *Ann N Y Acad Sci* **732**: 42–50

Muller D, Wolf C, Abecassis J, Millon R, Engelmann A, Bronner G, Rouyer N, Rio MC, Eber M and Methlin G (1993) Increased stromelysin 3 gene expression is associated with increased local invasiveness in head and neck squamous cell carcinomas. *Cancer Res* **53**: 165–169

Murray G, Duncan M, O'Neil P, Melvin W and Fothergill J (1996) Matrix metalloproteinase-1 is associated with poor prognosis in colorectal cancer. *Nature Med* **2**: 461–462

Naylor MS, Stamp GW, Davies BD and Balkwill FR (1994) Expression and activity of MMPS and their regulators in ovarian cancer. *Int J Cancer* **58**: 50–56

Newell KJ, Witty JP, Rodgers WH and Matrisian LM (1994) Expression and localization of matrix-degrading metalloproteinases during colorectal tumorigenesis. *Mol Carcinogen* **10**: 199–206

Nomura H, Sato H, Seiki M, Mai M and Okada Y (1995) Expression of membrane-type matrix metalloproteinase in human gastric carcinomas. *Cancer Res* **55**: 3263–3266

Okada Y, Gonoji Y, Naka K, Tomita K, Nakanishi I, Iwata K, Yamashita K and Hayakawa T (1992) Matrix metalloproteinase 9 (92-kDa gelatinase / type IV collagenase) from HT1080 human fibrosarcoma cells. *J Biol Chem* **267**: 21712–21719

Page RC (1991) The role of inflammatory mediators in the pathogenesis of periodontal disease. *J Periodont Res* **26**: 230–242

Parsons SL, Watson S, Griffin N and Steele R (1996) An open phase I/II study of the oral matrix metalloproteinase inhibitor, marimastat in patients with inoperable gastric cancer (abstract). *Ann Oncol* **7**: 47

Parsons SL, Watson S, Brown P, Collins H and Steele R (1997) Matrix metalloproteinases. *Br J Surg* **84**: 160–166

Pyke C, Ralfkiaer E, Tryggvason K and Dano K (1993) Messenger RNA for two type IV collagenases is located in stromal cells in human colon cancer. *Am J Pathol* **142**: 359–364

Sang QX, Birkedal-Hansen H and Van Wart HE (1995) Proteolytic and non-proteolytic activation of human neutrophil progelatinase B. *Biochim Biophys Acta* **1251**: 99–108

Sato H, Takino T, Okada Y, Cao J, Shinagawa A, Yamamoto E and Seiki M (1994) A matrix metalloproteinase expressed on the surface of invasive tumour cells. *Nature* **370**: 61–65

Satoh K, Ohtani H, Shimosegawa T, Koizumi M, Sawai T and Toyota T (1994) Infrequent stromal expression of gelatinase A and intact basement membrane in intraductal neoplasms of the pancreas. *Gastroenterology* **107**: 1488–1495

Sier C, Kubben F, Ganesh S, Heerding MM, Griffoen G, Hanemaaijer R, van Krieken JHJM, Lamers CBHW and Verspaget HW (1996) Tissue levels of MMP-2 and MMP-9 are related to the overall survival of patients with gastric carcinoma. *Br J Cancer* **74**: 413–417

© Cancer Research Campaign 1998

Suzuki K, Enghild JJ, Morodomi T, Salvesen G and Nagase H (1990) Mechanisms of activation of tissue procollagenase by matrix metalloproteinase 3 (stromelysin). *Biochemistry* **29**: 10261–10270

Talhouk R, Bissel M and Werb Z (1992) Co-ordinated expression of extracellular matrix degrading proteinases and their inhibitors regulates mammary epithelial function during involution. *J Cell Biol* **118**: 1271–1282

Wilson MJ and Sinha AA (1993) Plasminogen activator and metalloprotease activities of Du-145, PC-3, and 1-LN-PC-3-1A human prostate tumors grown in nude mice: correlation with tumor invasive behavior. *Cell Mol Biol Res* **39**: 751–760

Woessner JF, Jr (1995) Quantification of matrix metalloproteinases in tissue samples. *Methods Enzymol* **248**: 510–528

Wolf C, Chenard MP, Durand de Grossouvre P, Bellocq JP, Chambon P and Basset P (1992) Breast-cancer-associated stromelysin-3 gene is expressed in basal cell carcinoma and during cutaneous wound healing. *J Invest Dermatol* **99**: 870–872

Wysocki AB, Staiano-Coico L and Grinnell F (1993) Wound fluid from chronic leg ulcers contains elevated levels of metalloproteinases MMP-2 and MMP-9. *J Invest Dermatol* **101**: 64–68

Yamagata S, Yoshii Y, Suh JG, Tanaka R and Shimizu S (1991) Occurrence of an active form of gelatinase in human gastric and colorectal carcinoma tissues. *Cancer Lett* **59**: 51–55

Zeng ZS and Guillem JG (1995) Distinct pattern of matrix metalloproteinase 9 and tissue inhibitor of metalloproteinase 1 mRNA expression in human colorectal cancer and liver metastases. *Br J Cancer* **72**: 575–582

© Cancer Research Campaign 1998